# Nasal Microbiome in Granulomatosis with Polyangiitis Compared to Chronic Rhinosinusitis

**DOI:** 10.3390/diagnostics14151673

**Published:** 2024-08-02

**Authors:** Eliza Brożek-Mądry, Zofia Burska, Katarzyna Życińska, Janusz Sierdziński

**Affiliations:** 1 Department of Otorhinolaryngology, National Institute of the Ministry of the Interior and Administration, 02-507 Warsaw, Poland; 2 Department of Rheumatology, Connective Tissue Diseases and Rare Diseases, National Institute of the Ministry of the Interior and Administration, 02-507 Warsaw, Poland; 3 Department of Medical Informatics and Telemedicine, Medical University of Warsaw, 00-581 Warsaw, Poland

**Keywords:** granulomatosis with polyangiitis, microbiome, nasal septal perforation, chronic rhinosinusitis

## Abstract

Rhinosinusitis in granulomatosis with polyangiitis (GPA) is categorised as a secondary, diffuse and inflammatory chronic rhinosinusitis (CRS). It is one of the conditions that impacts the nasal microbiota. This study aimed to compare the nasal microbiomes of patients with GPA, CRS and NSP. A total of 31 patients were included in the study (18 GPA, 6 CRS and 7 nasal septum perforation (NSP)). In all patients, SNOT 22, a nasal endoscopy (Lund–Kennedy scale) and a brush swab were performed. The metagenomic analysis was carried out based on the hypervariable V3-V4 region of the 16S rRNA gene. At the genus level, statistically significant differences were observed in two comparisons: the GPA/NSP and the GPA/CRS groups. In the GPA/NSP group, the differences were related to four genera (*Actinomyces*, *Streptococcus, Methylobacterium-Methylorubrum*, *Paracoccus*), while in the GPA/CRS group, they were related to six (*Kocuria, Rothia, Cutibacterium, Streptococcus, Methylobacterium-Methylorubrum*, *Tepidimonas*). Patients with GPA had lower diversity compared to CRS and NSP patients. There were no statistically significant differences found for the *Staphylococcus* family and *Staphylococcus aureus* between the three groups.

## 1. Introduction

The human nasal microbiome is shaped from childbirth and plays a part in developing nasal mucosa functions and local immunity [1]. Dysbiosis (of the nasal microbiome) affects the pathogenesis of several conditions, such as chronic rhinosinusitis (CRS), asthma and allergic rhinitis. Bacteria perform structural and metabolic functions by regulating the work of the epithelium of the nose and paranasal sinuses, strengthening cell connections and taking part in metal sequestration, vitamin synthesis and fermentation of undigested polysaccharides and mucus [2,3,4,5]. Studies show that the nasal microbiome plays a significant role in developing upper respiratory tract infections (URTI) by affecting local immunity.

Sinonasal symptoms are the most common manifestation of granulomatosis with polyangiitis (GPA) in the head and neck region. GPA is an autoimmune disease characterised by antineutrophil cytoplasm antibodies (ANCA) involved in damaging the endothelium of small/medium vessels leading to necrosis [6,7,8]. According to the new European Position Paper on Rhinosinusitis and Nasal Polyps 2020 (EPOS 2020) guidelines, GPA is categorised as a secondary, diffuse and inflammatory CRS [9]. It is one of the conditions that disturb the nasal microbiota. GPA patients present changes, such as sinus opacification, bone/cartilaginous destruction, neoosteogenesis or septum perforation, and the most common nasal symptoms are epistaxis, nasal obstruction, discharge and crust formation [10,11]. The organs most frequently affected by GPA are kidneys, lungs and the upper respiratory tract, although any area can be involved.

Several studies focusing on the role of *Staphylococcus aureus* (*S. aureus*) in GPA have been published, although they were based on bacterial cultures. Patients with GPA are believed to be chronic carriers of *S. aureus*, more so than healthy populations [12,13]. Laudien et al. showed that patients with *S. aureus* in their nasal cavities had significantly higher endoscopically proven endonasal disease activity than non-Staphylococcus aureus carriers [13]. Moreover, patients with chronic *S. aureus* nasal colonisation are more prone to GPA relapse [12,13,14,15]. In addition, treatment with co-trimoxazole reduces the risk of relapse in patients with GPA [16,17]. However, other studies have questioned whether co-trimoxazole therapy and *S. aureus* carriage modify the risk of relapse [15,16,17,18]. Furthermore, *S. aureus* is considered to be a stimulant of chronic immune response in CRS [19].

The aim of the study was to analyse the microbiomes of patients with GPA and to compare them with patients with CRS and nasal septum perforation (NSP).

## 2. Materials and Methods

A prospective clinical study was conducted between May 2018 and May 2021. The study was approved by the Bioethical Committee (KB/198/2015). Analysed specimens were collected from patients treated and followed up in the Department of Otorhinolaryngology. We collected specimens from patients with GPA, NSP and CRS. All patients provided written informed consent. The GPA group involved 18 patients aged 24–78 (mean age 46.7; median 45.5), with a male-to-female ratio of 8:10. The group with CRS consisted of 6 patients aged 32–83 (mean age 50; median 42), with a male-to-female ratio of 2:4. The group with NSP consisted of 7 patients aged 25–65 (mean age 43; median 41), with a male-to-female ratio of 2:5. None of them were treated with antibiotics at the time of the nasal swab or 4 weeks before.

The GPA group characteristics included the Birmingham Vasculitis Activity Score (BVAS) V3.0. All patients filled out SNOT-22 (Sinonasal Outcome Test-22) [20]. The examination of the patients included nasal endoscopy evaluated with the Lund–Kennedy [21] endoscopic scoring system. Patients with GPA were, at the time of the study, treated with cyclophosphamide (6 patients), azathioprine (4 patients), methotrexate (4 patients), mycophenolate mofetil (1 patient), rituximab (1 patient) and glucocorticoids (1 patient). One patient received no medication. Patients with CRS received topical nasal steroids and nasal rinsing, while patients with NSP received lubricants.

A total of 31 patients had endoscopically guided sterile nasal swabs taken from the middle meatus or the area of the lateral nasal wall in case of necrotic changes in the middle turbinate in GPA patients. The swab was obtained with the use of eNAT (Copan Italia S.p.A)—a 1 mL preservation medium for nucleic acids. The material was stored at a temperature of −80 °C.

### 2.1. Metagenomic Analysis

The metagenomic analysis of the population of bacteria and archaea was carried out based on the hypervariable V3–V4 region of the 16S rRNA gene. The analysis was presented at the kingdom, phylum, class, order, family, genus and species levels. Specific sequences of primers 341F and 785R (16S analysis) were used to amplify the selected region and prepare the library. PCR was performed with Q5 Hot Start High-Fidelity 2X Master Mix, and the reaction conditions followed the manufacturer’s recommendations. Sequencing was carried out on the MiSeq apparatus, in the paired-end (PE) technology, 2 × 300 nt, using the Illumina v3 kit.

Automatic preliminary data analysis was performed on the MiSeq sequencer using the MiSeq Reporter (MSR) v2.6 software. The analysis consisted of the 2 following stages:

1. Automatic sample demultiplexing;
2. Generating FASTQ files containing raw reads.

Bioinformatic analysis ensuring the classification of readings to the species level was carried out with the QIIME 2 software package based on the Silva 138 reference sequences. The DADA2 package was also used to identify biological sequences from those newly created in the sequencing process. This package was also used to highlight unique sequences of biological origin (i.e., ASV [amplicon sequence variant]). The analysis consisted of the following stages.

1. Checking the quality of the readings:
  - Analysing the error profile of individual samples and the dynamic generation of parameters for quality control (FIGARO tool);
  - Carrying out a quality control based on the coefficient of the maximum expected errors of the sample.
2. Preprocessing data using the Cutadapt tool:
  - Removing adapter sequences;
  - Rejecting too short readings (a minimum length of 30 nt).
3. Selecting unique ASV sequences (using the DADA2 package) by the following process:
  - Filtering out the sequences containing errors created during the sequencing process (denoising);
  - Combining paired readings—to increase the accuracy of sequencing, they are performed in paired-end mode so that at later stages of the analysis, the corresponding forward and reverse readings must be combined;
  - Merging of identical, unique sequences while maintaining the number of springs and the quality profile (dereplicating);
  - Removing constructs resulting from incorrect sequence assembly during PCR (filtering chimeras).
4. Assigning a taxonomy to the ASV sequence based on the Silva reference base and using a hybrid approach:
  - Comparing ASV sequences against the base in search of identical reference sequences (VSEARCH);
  - Classifying the atypical sequences remaining after the previous step based on methods using machine learning (sklearn).

### 2.2. Statistical Analysis

Statistical analysis included comparing quantitative variables by U test (Mann–Whitney). The median values of bacterial abundance were calculated using the Mann–Whitney test. The mean values were used to show mean abundance. The graphs showing mean abundance were prepared using an Excel sheet. To evaluate bacterial communities, alpha diversity was assessed. The Shannon diversity index measured alpha diversity (within-sample), which accounts for the evenness and richness (number) of the ASVs within a sample.

In all the calculations, the statistical significance level was assumed to be a value of *p* ≤ 0.05. The calculations were performed using Statistical Analysis System (SAS) software 94 from the SAS Institute, Inc. (Cary, NC, USA) and an Excel spreadsheet 2021 from Microsoft^®^.

## 3. Results

In the GPA group, the mean scores obtained by patients in the SNOT22 questionnaire were 33.86 (range 11–66); for the CRS and NSP groups, the mean scores were 30.50 (range 19–52) and 41.29 (range 12–67), respectively. The Lund–Kennedy scale mean scores were 6.11 (range 1–11), 5.75 (range 4–7) and 5.14 (range 4–7) for GPA, CRS and NSP patients, respectively. Nasal crusting results were as follows: 1.5 (range 0–4); 1.58 (range 1–3); and 2.21 (1–3) for the GPA, CRS and NSP groups, respectively. BVAS was only assessed in the GPA group with a mean score of 4.36 (range 1–10). There were no statistical differences between groups regarding these parameters.

**Figure 1:**
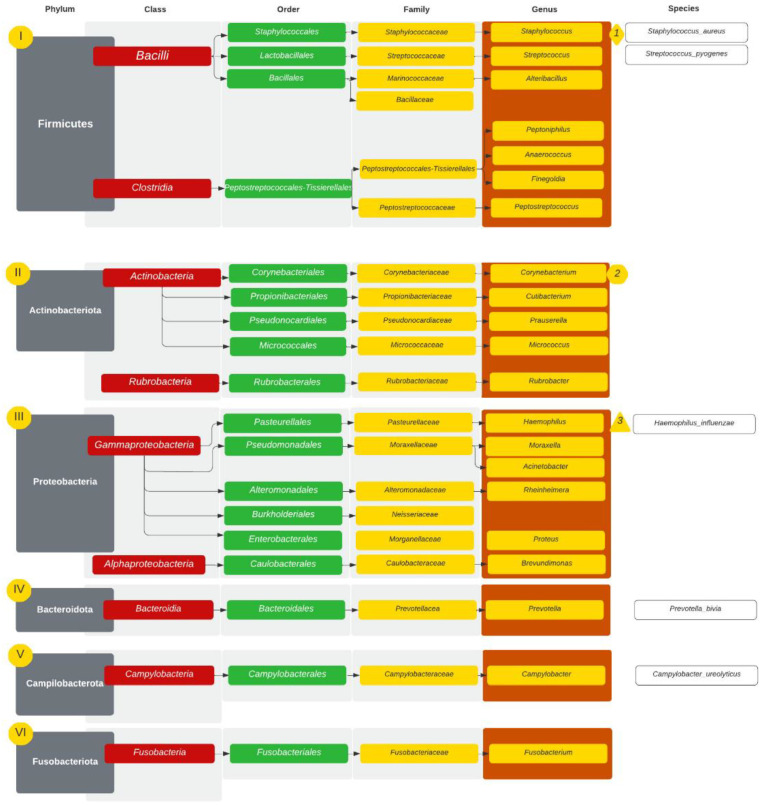
Taxonomic ranks in GPA patients with 3 most abundant genera marked. I–VI—incidence of phyla starting with I—most common.

**Figure 2:**
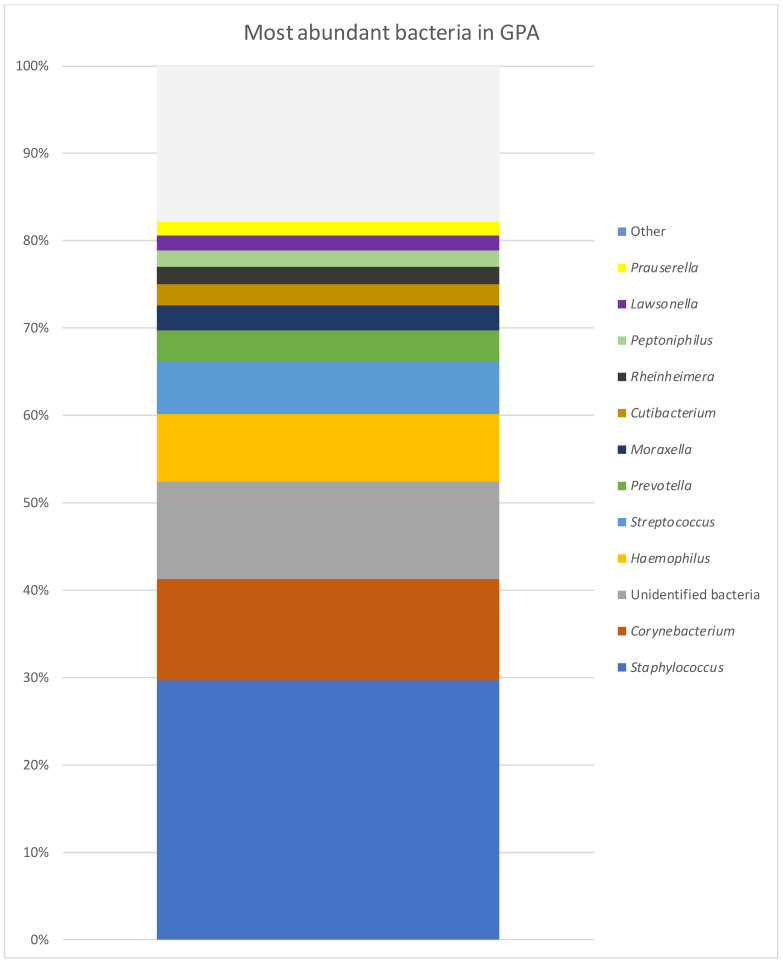
Most abundant bacteria in GPA group at genus level.

The microbiomes were analysed in 31 patients (18 GPA, 6 CRS and 7 NSP). Among patients with GPA (n = 18), at the levels of phylum to genus, bacteria were unidentified in more than 50% of a single microbiome in four patients, who were further excluded from the analysis. Figure 1 presents the most common taxonomic ranks in GPA patients. The most common taxonomic ranks belonged to Firmicutes, Actinobacteriota and Proteobacteria. In 14 patients with GPA at the genus level, the microbiome presented 184 different bacterial genera. The diversity at the genus level observed in those patients ranged between 15 and 67 (mean value 31). Five genera were found in every sample: *Staphylococcus*, *Corynebacterium*, *Cutibacterium*, *Prauserella* and *Rubrobacter*. The genera were observed in higher abundance but only in some microbiomes: *Streptococcus* (9), *Haemophilus* (7) and *Prevotella* (7). In lower abundances but commonly appearing in the microbiome *were Lawsonella* (10), *Alteribacillus* (10), *Finegoldia* (9), *Micrococcus* (8) and *Prevotella* (7). The microbiome with the most abundant genera is presented in Table 1 and Figure 2. At the species level, *S. aureus* was found in 12 of the 14 samples (with a relative abundance of 0–69% and a mean value of 26.35%), and it was the most common bacteria in the study group. The following were *Corynebacterium* sp. (11.34%), *Haemophilus influenzae* (*H. influenzae*) (7.42%), *Streptococcus pyogenes* (*S. pyogenes*) (5.35%), *Staphylococcus* sp. (2.98%), *Moraxella* sp. (2.77%), *Cutibacterium* sp. (2.46%), *Prevotella* sp. (1.88%), *Prauserella* sp. (1.67%), *Rheinheimera* sp. (1.39%), *Rubrobacter* sp. (1.39%), *Alteribacillus* sp. (1.05%) and *Prevotella bivia* (*P. bivia*) (1.01%). *Staphylococcus epidermidis* (*S. epidermidis*) was found in two patients (mean abundance 0.05%) and *Staphylococcus lugdunensis* (*S. lugdunesis*) was present in one GPA patient microbiome.

**Figure 3:**
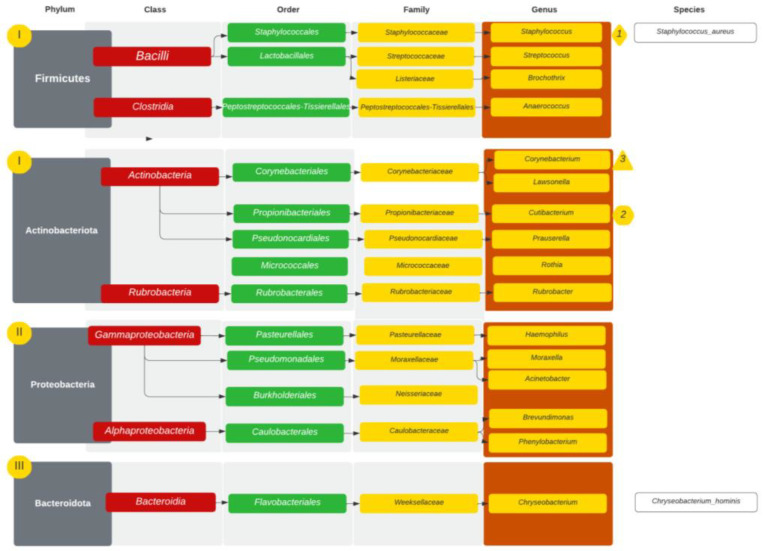
Taxonomic ranks in CRS patients with 3 most abundant genera marked. I–III—incidence of phyla starting with I—most common (ex aequo Firmicutes and Actinobacteriota).

**Figure 4:**
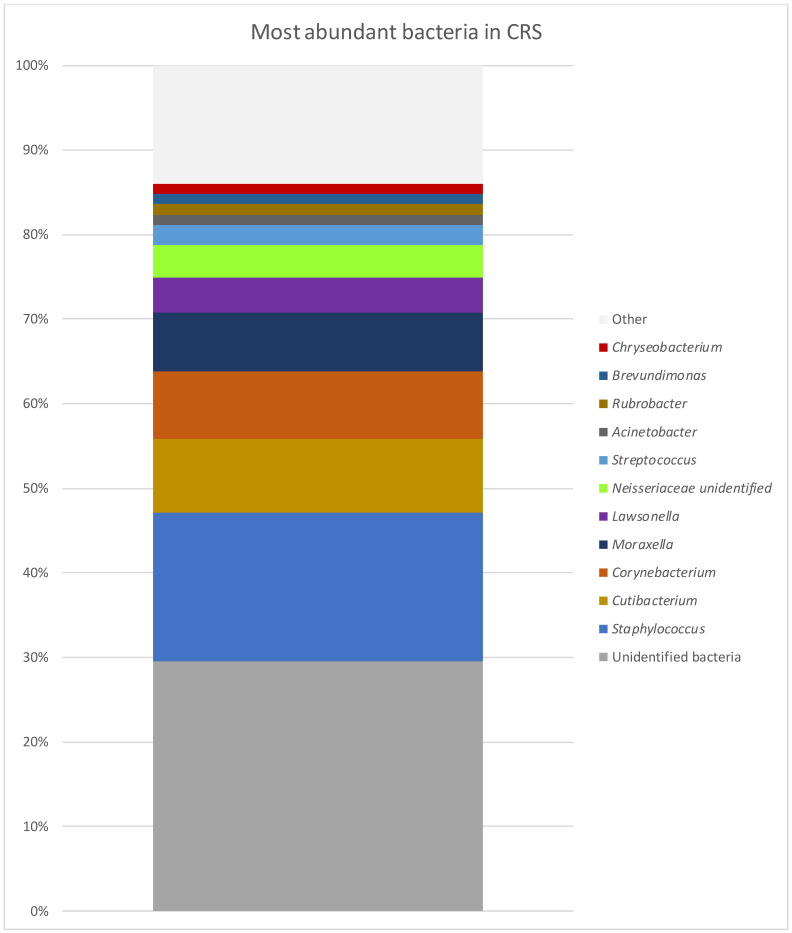
Most abundant bacteria in CRS group at genus level.

The most common taxonomic ranks in the CRS group (6) are presented in Figure 3. In those patients, the microbiome presented with 207 different bacterial genera. The microbiome with the most abundant genera is presented in Table 1 and Figure 4. The most common taxonomic ranks belonged to Firmicutes and Actinobacteria, Proteobacteria and Bacteroidota. The differences in diversity between the samples ranged between 44 and 65 (mean value 53). The genera found in every sample were *Staphylococcus*, *Cutibacterium*, *Corynebacterium*, *Brevundimonas*, *Streptococcus*, *Rubrobacter*, *Brevundimonas* and *Anaerococcus. Lawsonella* 4/6 samples (mean abundance 4.10%) and *Acinetobacter* 5/6 samples (mean abundance 1.27%) were found in high abundances. *Moraxella* was obtained only in a single sample but in a high abundance of 42.28%. At the species level, *S. aureus* was found at 11.89% abundance, followed by *Cutibacterium* sp. (8.55%) and *Corynebacterium* sp. (7.71%). *S. epidermidis* was found at 0.3%.

**Figure 5:**
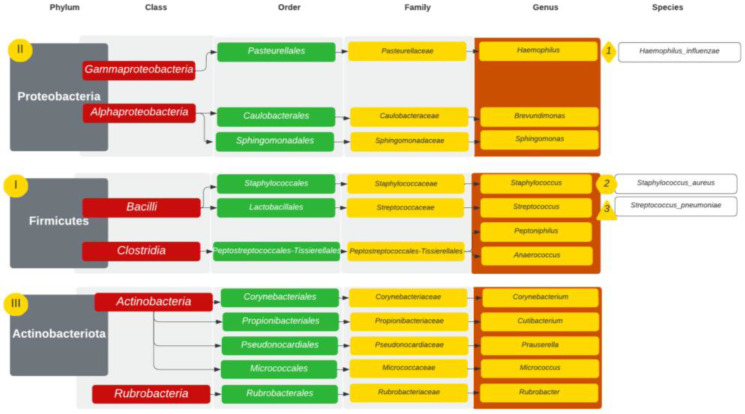
Taxonomic ranks in NSP patients with 3 most abundant genera marked. I–III—incidence of phyla starting with I—most common.

**Figure 6:**
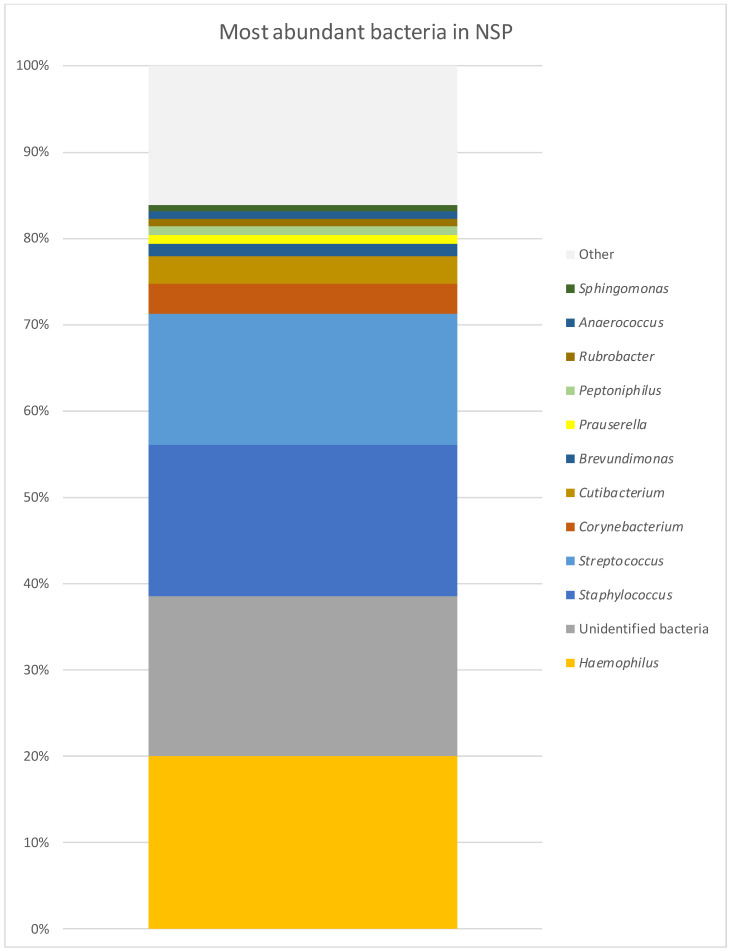
Most abundant bacteria in NSP group at genus level.

In patients with NSP (7), the taxonomic ranks belonged to Proteobacteria, Firmicutes and Actinobacteriota. The most common taxonomic ranks are presented in Figure 5. The diversity of bacterial genera was 309. In a single sample, bacterial diversity ranged between 20 and 228 (mean value 77). The genera obtained in every sample were *Staphylococcus, Streptococcus, Corynebacterium*, *Cutibacterium*, *Prauserella* and *Rubrobacter*. In six of the seven microbiome samples, there were *Brevundimonas*, *Lawsonella*, *Paracoccus*, 5/7—*Peptoniphilus*, *Anaerococcus*, *Sphingomonas*, *Micrococcus*, *Rothia*, *Veilonella*, *Phenylobacterium* and *Enhydrobacter*. The microbiome with the most abundant genera is presented in Table 1 and Figure 6. At the level of species, the most common was *H. influenzae*, which was present in high abundance but only in two samples (42.29%; 77.47%), and the next was *S. aureus*, which was present in six of the seven samples, with relative abundance ranging from 0 to 54.16%, with a mean of 13.85%. These were followed by *Streptococcus pneumoniae (S. pneumoniae)* (10.58%), other *Streptococcus* sp. (3.52%), *Staphylococcus* sp. (3.39%), *Cutibacetrium* sp. (3.19%), *Corynebacterium* sp. (2.67%), *Haemophilus* sp. (2.64%), *Brevundimonas* sp. (1.34%) and *Prauserella* sp. (1.04%).

In the GPA group, the microbiome presented 106 different bacterial families. The families found in every sample were *Staphylococcaceae*, *Corynebacteriaceae*, *Pseudonocardiaceae*, *Rubrobacteriaceae* and *Propionibacteriaceae.* Seven families were present in higher abundance but only in some samples: *Streptococcaceae* (10), *Pasteurellaceae* (7), *Moraxellaceae* (7), *Prevotellaceae* (7), *Alteromonadaceae* (2), *Marinococcaceae* (12) and *Peptostreptococcales-Tissierellales* (9). The family with the highest mean abundance was *Staphylococcaceae* (mean 29.67%, 1.42–72.30%).

In the CRS group, 99 bacterial families were observed. In every sample we found *Staphylococcaceae*, *Corynebacteriaceae*, *Moraxellaceae*, *Probinibacteriaceae*, *Neisseriaceae*, *Streptococcaceae*, *Caulobacteraceae*, *Peptostreptococcales-Tissierellales*, *Micrococcaceae*, *Weeksellaceae*, *Rubrobacteriaceae*, *Comamonadaceae*, *Marinococcaceae* and *Pseudonocardiaceae*. The following families were present among five out of six samples: *Pasteurellaceae*, *Bacillaceae*, *Beijerinckiaceae* and *Leptotrichiaceae*, while *Lactobacillaceae*, *Xanthobacteraceae*, *Actinomycetaceae* were observed in four out of six patients. The family with the highest mean abundance was *Staphylococcaceae* (mean 17.47%, 1.79–54.70%).

In the NSP group, the microbiome contained 166 families. The families obtained in every sample were *Staphylococcaceae*, *Streptococcaceae*, *Corynebacteriaceae*, *Probinibacteriaceae, Micrococcaceae*, *Caulobacteraceae*, *Pseudonocardiaceae*, *Rubrobacteriaceae* and *Marinococcaceae*. Two families were found in higher abundance but not in every sample: *Pasteurellaceae* and *Peptostreptococcales-Tissierellales*. The family with the highest mean abundance was *Pasteurellaceae* (mean 20.13%, 0.00–94.91%). Six families were observed in six out of seven samples: *Rhodobacteraceae*, *Bacillaceae*, *Actinomycetaceae*, *Flavobacteriaceae* and *Beijerinckiaceae*. In five out of seven patients we found *Sphingomonadaceae*, *Moraxellaceae*, *Comamonadaceae*, *Veillonellaceae*, *Weeksellaceae* and *Neisseriaceae*.

The differences between the analysed groups are presented in Table 2 at the family level. Statistically significant differences were observed in all three possible comparisons: the GPA/NSP, GPA/CRS and NSP/CRS groups. In the first two groups, the differences were present for seven families, while NSP/CRS differed in only two families.

The analysis in the GPA group was made for BVAS lower than five and higher than five and showed no statistically significant differences.

The differences at the genus level in the analysed groups are presented in Table 3. Statistically significant differences were observed in two comparisons: the GPA/NSP and GPA/CRS groups. In the GPA/NSP group, the differences were related to four genera, while in the GPA/CRS group, they were related to six. Comparing GPA and CRS showed lower median abundance in all groups except for *Streptococcus*, which was observed at higher abundance in GPA patients. There were no statistically significant differences between NSP and CRS groups.

**Figure 7:**
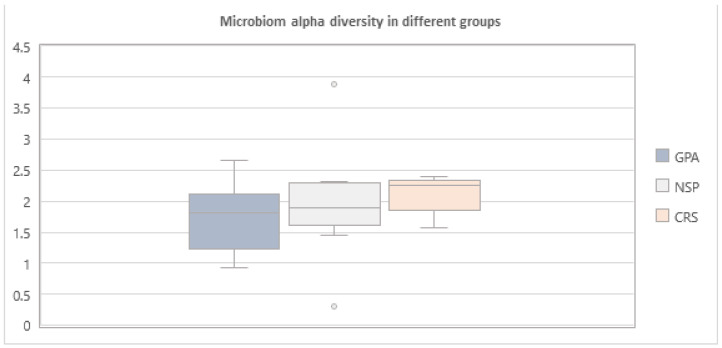
Alpha diversity in the 3 analysed groups—GPA, NSP and CRS.

The alpha diversity was the highest in CRS group (Figure 7).

## 4. Discussion

This study presents three groups of patients and their nasal microbiomes, and concentrates on a group of patients with GPA during disease treatment (cyclophosphamide/azathioprine/methotrexate). A few papers examining nasal microbiomes in GPA patients have been published [22,23,24,25]. In this paper, we aimed at finding differences between the nasal microbiomes of patients with GPA and two other groups: primary CRS and NSP. GPA commonly presents with rhinosinusitis symptoms and is categorised as secondary CRS. According to the internationally accepted Chapel Hill Consensus Nomenclature, GPA is defined as an ANCA-associated vasculitis, characterized by necrotizing granulomatous inflammation usually involving the upper and lower respiratory tract, and necrotizing vasculitis predominantly affecting small to medium vessels (e.g., capillaries, venules, arterioles, arteries and veins), in which necrotizing glomerulonephritis is common [7,26]. Moreover, NSP can be one of the symptoms in head and neck GPA, and when it is observed, GPA should be considered in a differential diagnosis. Interestingly, the most severe symptoms (highest SNOT-22 score) were observed in NSP patients. We attribute this to the fact that both our GPA and CRS patients were treated, while the NSP patients remained without treatment. It also corresponded with the most severe nasal crusting in the NSP group, rather than GPA.

Studies evaluating the microbiome using culture-independent sequencing technology in GPA have already been published. In a study by Rhee et al., 60 GPAs and 41 healthy controls were included [23]. The nasal microbiome was assessed using 16S rRNA gene sequencing. The most abundant species identified in all participants were *Corynebacterium tuberculostearicum* (*C. tuberculostearicum*), *Propionibacterium acnes* (*P. acnes*), *S. aureus and S. epidermidis. P. acnes and S. epidermidis* had a significantly lower abundance in patients with GPA compared to controls, while no difference in the abundance of *S. aureus* was observed. Moreover, the amount of nasal microbiota in patients with GPA who received non-glucocorticoid immunosuppression was similar to the healthy controls, whereas in patients with GPA who were not taking non-glucocorticoid immunosuppression, the amount was significantly different. Patients without immunosuppression had a significantly lower abundance of *Propionibacterium* compared to controls, while participants with GPA immunosuppression had an abundance similar to controls [23]. In the present study, *Propionibacteriaceae* differed significantly in GPA patients with immunosuppression and patients with primary CRS, showing lower abundance in GPA, similarly to the work by Rhee et.al. Considering *Staphylococcaceae*, even though mean abundance was higher in patients with GPA, statistical analysis did not show significant differences compared to NSP or CRS. Overall, analysis of the lower abundance of *Cutibacterium* (mean 2,49% in GPA and 8,80% in CRS) belonging to the family *Propionibacteriaceae* and the higher *Staphylococcus* load in GPA patients nasal microbiome (mean 29,65% and 17,47% in CRS) showed that they could be explained by the decreased production of agents exhibiting anti-Staphylococcal properties attributed to *Cutibacterium* [27,28,29]. The differences at the species level also contribute to *S. aureus* promotion in GPA. The abundances of *S. epidermidis* and *S. lugdunensis* were lower in GPA when compared to CRS. Li et al. [30] demonstrated in vitro that lipopeptide from *S. epidermidis* increases the expression of human β-defensin 2 (hBD2) and hBD3 on keratinocytes in neonates, which consequently leads to the inhibition of *S. aureus* growth. They also found a role for lipopeptide in increasing the expression of mouse β-defensin 4 via a toll-like receptor 2 (TLR2-dependent) pathway. Another nasal commensal, *S. lugdunensis*, produces lugdunin, an antibiotic that is bactericidal for *S. aureus* and *S. pneumoniae*—carriage of *S. lugdunensis* correlates with reduced *S. aureus* in people [31]. In the study by Zipperer et al., the authors describe a new antibiotic called lugdunin produced by the commensal *S. lugdunensis* that is bactericidal for *S. aureus* in vitro and reduces *S. aureus* growth in a rat nasal colonization model. *S. lugdunensis* is also negatively associated with *S. aureus* colonization in humans.

Lamprecht et al. evaluated nasal microbiota in GPA using 16S rRNA sequencing [25]; the study involved 29 GPA patients, 21 disease controls (rheumatoid arthritis [RA]) and 27 healthy controls. The nasal microbiome in GPA mainly contained three phyla: Proteobacteria, Firmicutes and Actinobacteria. This did not differ at the phylum level among samples from healthy and disease controls and GPA patients. In our study, the most common phylum in both disease controls and GPA was Firmicutes, followed by Actinobacteriota only in GPA patients. In Lamprecht et al.’s work, significant differences in the bacterial composition were observed at the class, family and species levels. In GPA and RA patients, abundance of the bacterial family *Planococcaceae* increased. In addition, the bacteria species included in the families *Streptococcaceae*, *Pasteurellaceae* and *Prevotellaceae* also increased in the GPA group. In our group, the same observation was made with *Prevotellaceae*, but for *Streptococcaceae* the abundance was highest in NSP. In Lamprecht et al.’s GPA group, the abundance of species from the families *Corynebacteriaceae*, *Moraxellaceae, Tissierellaceae*, *Staphylococcaceae* and *Propionibacteriaceae* decreased. The relative abundance of *Porphyromonas* was not detected in GPA patients, but it was present at low levels in the group of RA patients and controls. The authors also compared the microbiomes of patients with active GPA and GPA in remission. Patients with active GPA had similar microbiomes at the phylum, class and family levels, except for *Staphylococcaceae*, which was more abundant in patients in remission. In the GPA group, the bacteria composition depends on the severity of ENT symptoms. Patients with nasal symptoms had increased abundance of the *Streptococcaceae* and *Planococcaceae* families and a reduced abundance of *Corynebacteriacea*. Among RA patients, no major differences were found between the active disease and remission groups.

In contrast to the previously mentioned study by Rhee et al. [23], *S. aureus* was significantly more abundant in patients with GPA than the disease and healthy controls. Similar tendencies were observed in the case of *H. influenzae*, yet the results did not reach the level of significance. In our comparison of GPA and CRS, significant differences were observed in families such as *Propionibacteriaceae*, *Weeksellaceae*, *Caulobacteraceae*, *Beijerinckiaceae*, *Xanthobacteraceae*, *Sphingomonadaceae* and *Moraxellaceae*. In GPA, they had much lower abundances. When we compared GPA to NSP, significant differences were observed in the following families: *Actinomycetaceae*, *Flavobacteriaceae*, *Weeksellaceae*, *Streptococcaceae*, *Lachnospiraceae*, *Beijerinckiaceae* and *Rhodobacteraceae.* Similarly, the abundance of all families was higher in NSP patients.

The nasal cavity is constantly interacting with the external environment, possibly leading to high microbial diversity in nasal niches. Lamprecht et al. mentioned that in GPA, there is a trend toward lower alpha diversity in the nasal microbiome of GPA samples compared with the control samples [25]. In our study, the alpha diversity in GPA patients was the lowest and in primary CRS patients it was the highest. The low alpha diversity in patients with GPA may be due to recurrent antibiotic treatment prior to the diagnosis and may also be attributed to the immunosuppressive treatment. Rhee et al. found no significant differences in Shannon diversity between patients with relapsing vs. non-relapsing disease; also, no difference was observed in the diversity measures when comparing remission, pre-relapse, relapse and post-relapse visits [22].

In a case–control study conducted by Wagner et al. [24] on 12 patients with active GPA, 44 with inactive GPA, 13 disease controls and 15 healthy controls, nasal microbiota was assessed using bacterial culture and 16S rRNA. In the nasal culture, *S. aureus* was significantly more frequently detected in patients with active GPA (66.7%) than with inactive GPA (34.1%). In 16 S rRNA analysis, patients with GPA (active and inactive groups) had significantly different compositions of nasal microbiota compared to controls. In addition, shotgun metagenomic sequencing was conducted in this study and was focused on *Staphylococcus* taxa, since it was the most abundant one in the study. *S. aureus* was significantly more abundant in patients with active GPA than in disease and healthy controls, while *S. epidermidis* was more abundant in healthy controls. Moreover, *Staphylococcus pseudintermedius*, which is a species frequently observed in cats and dogs [32], was detected in all groups, although there were no significant differences between groups. Furthermore, in contrast to the previously mentioned thesis *S. aureus*, abundance was not associated with a higher risk of GPA relapse [24]. In this study, disease activity was assessed using the BVAS scale. We found no statistically significant differences in the nasal microbiomes of patients with higher and lower disease activity according to the BVAS scale.

A study by Rhee et al. investigated long-term changes in the nasal microbiota using 16S rRNA, in which patients were observed longitudinally. Nine participants experienced a GPA relapse. Across all nasal samples, *Corynebacterium* and *Staphylococcus* were the most abundant genera. Patients with stable GPA had a constant *Corynebacterium* to *Staphylococcus* ratio (C/S), while patients who first experienced a relapse underwent a decrease in the C/S ratio and then an increase. Rhee et al. found an association between a higher abundance of *C. tuberculostearicum* and relapse. Moreover, the authors found that an increasing abundance of nasal *C. tuberculostearicum* was associated with higher levels of PR3-ANCA [22]. In our patients, the C/S mean ratio was similar for GPA and CRS and we observed a decreased ratio in NSP patients—the group with the highest mean SNOT-22 score.

## 5. Conclusions

In conclusion, the study showed that there are some statistically significant differences in the nasal microbiomes of patients with GPA, CRS and nasal septum perforation. Moreover, patients with GPA had lower diversity compared to CRS and NSP patients. *Staphylococcus* mean abundance was highest in the GPA patients, but no statistically significant differences were found for *Staphylococcaceae* and *S. aureus* between the GPA and disease controls. *Cutibacterium* (*Propionibacterium*) was decreased in GPA patients when compared to disease controls. In NSP and GPA, the mean abundances of *Corynebacterium* and *Cutibacterium* were comparable. The decreased abundance of *Cutibacterium* may underlie higher *Staphylococcus* abundance.

## Figures and Tables

**Table 1:** The mean abundance of bacteria in patients with GPA, CRS and NSP at the genus level.

	GPA	CRS	NSP
*Staphylococcus*	29.65%	17.47%	17.42%
*Corynebacterium*	11.65%	7.93%	3.58%
Unidentified bacteria	11.16%	29.60%	18.47%
*Haemophilus*	7.70%	0.50%	20.03%
*Streptococcus*	5.97%	2.93%	15.16%
*Prevotella*	3.68%	0.12%	0.21%
*Moraxella*	2.77%	7.05%	0.32%
*Cutibacterium*	2.49%	8.80%	3.20%
*Rheinheimera*	1.95%	0.03%	0.01%
*Peptoniphilus*	1.84%	0.37%	1.01%
*Lawsonella*	1.71%	4.10%	0.48%
*Prauserella*	1.70%	0.50%	1.04%
*Alteribacillus*	1.56%	0.46%	0.23%
*Rubrobacter*	1.39%	1.26%	0.94%
*Anaerococcus*	1.06%	0.83%	0.75%
*Neisseriaceae unidentified*	0.69%	3.80%	0.01%
*Acinetobacter*	0.98%	1.27%	0.24%
*Brevundimonas*	0.59%	1.19%	1.35%
*Chryseobacterium*	0.00%	1.18%	0.22%

**Table 2:** Statistically significant differences between the groups at the level of the family: (**A**)—GPA/NSP; (**B**)—GPA/CRS; (**C**)—NSP/CRS.

(**A**)					
**Family**		**U Mann–Whitney Test**			
		**Median GPA**		**Median NPS**	***p*-Value**
***Actinomycetaceae***		0.00%		0.05%	0.016
***Flavobacteriaceae***		0.00%		0.04%	0.038
***Weeksellaceae***		0.00%		0.13%	0.016
***Streptococcaceae***		0.42%		11.22%	0.025
***Lachnospiraceae***		0.00%		0.39%	0.025
***Beijerinckiaceae***		0.00%		0.23%	0.020
***Rhodobacteraceae***		0.01%		0.32%	0.031
(**B**)					
**Family**			**U Mann–Whitney Test**		
			**Median GPA**	**Median CRS**	***p*-Value**
***Propionibacteriaceae***			1.15%	4.78%	0.026
***Weeksellaceae***			0.00%	0.18%	<0.001
***Caulobacteraceae***			0.25%	0.89%	0.041
***Beijerinckiaceae***			0.00%	0.14%	0.041
***Xanthobacteraceae***			0.00%	0.12%	0.041
***Sphingomonadaceae***			0.02%	0.28%	0.015
***Moraxellaceae***			0.02%	2.57%	0.033
(**C**)					
**Family**	**U Mann–Whitney Test**				
	**Median NPS**			**Median CRS**	***p*-Value**
***Lachnospiraceae***	0.39%			0.00%	0.035
***Neisseriaceae***	0.13%			0.78%	0.005

**Table 3:** Statistically significant differences between the groups at the genera level: (**A**)—GPA/NSP; (**B**)—GPA/CRS.

(**A**)			
**Genus**	**U Mann–Whitney Test**		
	**Median GPA**	**Median NSP**	***p*-Value**
***Actinomyces***	0.00%	0.03%	0.008
***Streptococcus***	0.17%	11.22%	0.008
***Methylobacterium-Methylorubrum***	0.00%	0.11%	0.014
***Paracoccus***	0.00%	0.32%	0.046
(**B**)			
**Genus**	**U Mann–Whitney test**		
	**Median GPA**	**Median CRS**	***p*-Value**
***Kocuria***	0.00%	0.17%	0.022
***Rothia***	0.00%	0.36%	0.017
***Cutibacterium***	1.15%	4.72%	0.036
***Streptococcus***	0.17%	1.64%	0.046
***Methylobacterium-Methylorubrum***	0.00%	0.14%	0.022
***Tepidimonas***	0.00%	0.25%	0.046

## Data Availability

The raw data supporting the conclusions of this article will be made available by the authors on request.
